# Primary pulmonary meningioma presenting as a pulmonary ground glass nodule: a case report and review of the literature

**DOI:** 10.1186/s13256-024-04668-z

**Published:** 2024-08-02

**Authors:** Shengliang Zhao, Xiaoqing Liu, Mingzhang Xiang, Jigang Dai

**Affiliations:** Department of Thoracic Surgery, Xinqiao Hospital, Army Medical University (Third Military Medical University), No. 183, Xinqiaozheng Street, Shapingba District, Chongqing, 400037 China

**Keywords:** Primary pulmonary meningioma, Pulmonary ground glass nodule, Thoracoscopic pulmonary wedge resection

## Abstract

**Background:**

A primary pulmonary meningioma is an extremely rare entity. Primary pulmonary meningiomas manifested with a ground glass nodule are a very rare occurrence in clinical practice.

**Case presentation:**

In this study, we report a case of a primary pulmonary meningioma with atypical computed tomography features. A 59-year-old Han Chinese female came to our hospital for treatment and reported that her physical examination revealed a ground glass nodule in the right lung for over 3 months. The histologic result revealed a primary pulmonary meningioma. The patient underwent a thoracoscopic lung wedge resection of the right upper lobe for a ground glass nodule. After 1 year of follow-up, the patient is still alive without evidence of metastasis or recurrence.

**Conclusions:**

Primary pulmonary meningiomas could have a variety of radiological findings. As there are no specific radiologic features for the diagnosis of primary pulmonary meningiomas, complete resection of the lesion is required for both diagnosis and treatment. It is necessary to note the imaging features of primary pulmonary meningiomas, presenting as a ground glass nodule; this rare tumor should be considered in differential diagnoses.

## Introduction

A meningioma is a common primary tumor in the central nervous system (CNS). An ectopic primary meningioma, which accounts for 1–2% of all primary meningiomas, is rare: they occur in several locations, such as the head-and-neck region, skin, bone, peripheral nerves, retroperitoneum, and lung [1, 2]. Primary pulmonary meningioma (PPM) is a very rare and mostly benign disease. Few previous studies have reported the clinical features of PPM, which has led to the low diagnostic rate of early PPM. Rates of misdiagnosis and missed diagnosis are relatively high in clinical practice. PPM manifested with a ground glass nodule (GGN) is a very rare occurrence in clinical practice. Recently, a patient with PPM and GGN was diagnosed and treated at our medical center. This paper describes this rare form of PPM through a case report and literature review.

## Case presentation

**Fig. 1:**
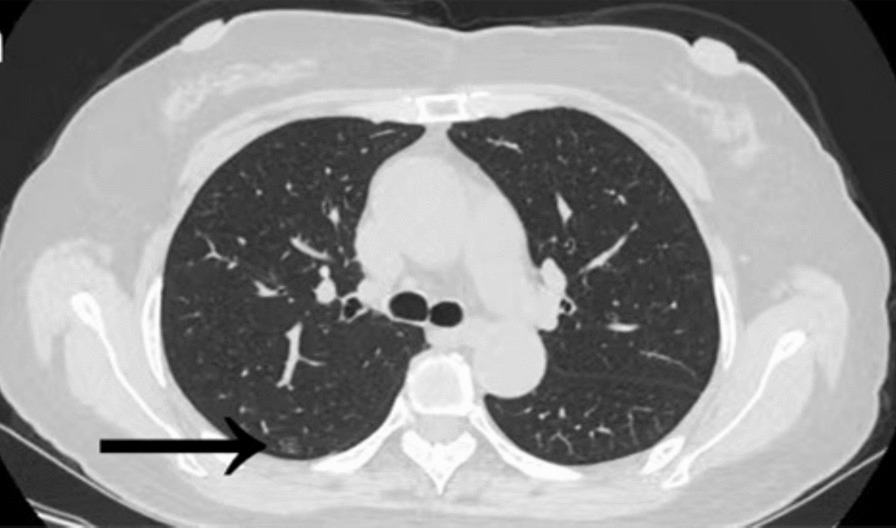
Chest CT scan showing a 4–5 mm GGN in the right upper lobe (arrowheads)

A 59-year-old Han Chinese female came to our hospital for treatment and reported that her physical examination revealed a GGN in the right lung for over 3 months. Chest computed tomography (CT) was performed at our hospital, revealing a 4.0–5.0 mm GGN in the right upper lobe (Fig. 1). Due to the small size and low density of the GGN, we first suspected it to be a pneumonia nodule. After anti-inflammatory treatment for half a month, the chest CT reexamination showed that the GGN still existed. The patient had no history of chronic disease, malignant tumor, smoking, tuberculosis, or cancer in the family. The results of the laboratory tests were within normal limits. The central nervous system (CNS) was normal on magnetic resonance imaging (MRI). Also, no abnormality in the central nervous system had been recognized.

**Fig. 2:**
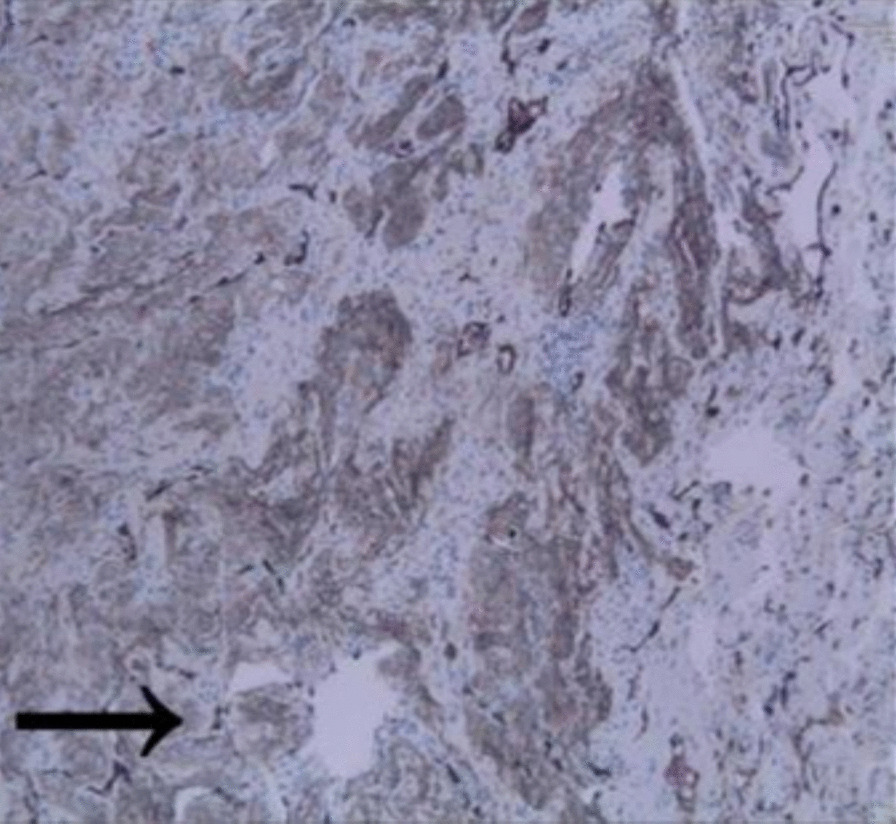
Immunohistochemically (IHC), the tumor cells show staining positive for epithelial membrane antigen (EMA), 200× (arrowheads)

**Fig. 3:**
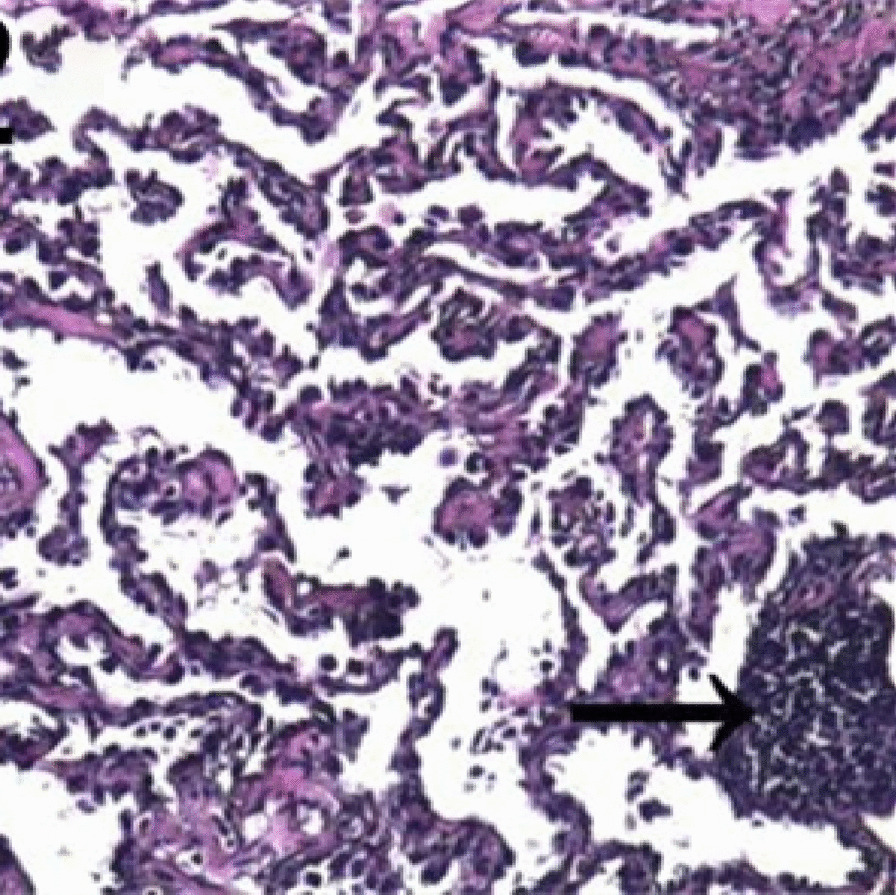
A medium-power photomicrograph of the tumor showing spindle-shaped cells with poorly defined cell borders arranged in whorls. Hematoxylin & eosin, 100× (arrowheads)

A thoracoscopic wedge resection of the GGN was performed with an intraoperative frozen section, revealing a spindle-cell tumor. Immunohistochemically, the tumor cells showed staining positive for epithelial membrane antigen (EMA) and vimentin, whereas they were negative for keratin, S-100 protein, and neuron-specific enolase (Fig. 2). A medium-power photomicrograph of the tumor showed spindle-shaped cells with poorly defined cell borders arranged in whorls (Fig. 3).

Finally, a histological diagnosis of PPM without characteristics of malignancy was made according to the morphological and immunohistochemical features described above. After 1 year of follow-up, the patient was still alive without evidence of metastasis or recurrence. The patient agreed to authorize us to share these figures and experiences during her treatment procedure in our department. Informed consent was obtained.

## Discussion

Reports of meningiomas that primarily originate outside the CNS are very rare. Thus far, these meningiomas have been detected in the following sites: intraorbital, scalp and subcutaneous tissue, skull, sinonasal, intraparenchymal, epidural, parotid, thorax, adrenal, and finger [3]. Meningiomas that primarily originate from the lung are more rare. There are several controversial hypotheses regarding the pathogenesis of PPM. For instance, some authors have proposed that the tumor develops from minute pulmonary meningothelial-like nodules [4], whereas others suggest that the tumor may arise from pluripotent subpleural mesenchyme [5]. The true etiology of this tumor is still uncertain [6]. A total of 68 patients diagnosed with PPM were reported in the English literature from 1982 to 2021 [7, 8].

PPM is a rare disease that is usually asymptomatic and primarily occurs in 40–60-year-old patients. Nevertheless, patients with larger tumors may experience chest pain and other symptoms [9]. In our case, the patient did not have any clinical symptoms, and the GGN was found during physical examination. In the vast majority of patients, pulmonary masses and nodules are detected during physical examination. X-rays and CT scans usually reveal round or oval solitary nodules or mass shadows with different sizes, uniform density, smooth boundaries, and a clear outline [10]. Previous reports of PPM with contrast-enhanced CT images showed various enhancement patterns, such as heterogeneous or nodular enhancement [11], poor enhancement [12], or homogeneous enhancement [7].

In our case, it is very rare for PPM patients to have a GGN on a chest CT. Because of its small size, low density, and blurry edge, we believed it to be a pneumonia node in the early diagnosis, but did not exclude the possibility of atypical adenomatous hyperplasia, adenocarcinoma in situ, and minimally invasive adenocarcinoma. We reviewed chest CT half a month after anti-inflammatory treatment, and the GGN was still present. The patient’s lung nodule was a sub-centimeter (4–5 mm) nodule. As per the guidelines and surveillance protocol, no surgical intervention was indicated. However, the patient was in a serious state of anxiety and depression, and we considered the patient to have “pulmonary nodule syndrome.” To alleviate the psychological state of anxiety and depression, we obtained the consent of the patient’s family, so we performed surgical resection, and the PPM was confirmed after surgery. Distinguishing PPM from other lung tumors may be difficult, as the most common manifestation is an isolated GGN. Therefore, the early diagnosis of such a GGN is difficult. Pathological identification is necessary for a final diagnosis of PPM. In many cases, an enhanced CT scan shows a mass with different degrees of non-uniform enhancement, distinguished from uniform and apparent enhancement in an intracranial meningioma [10]. Vimetin and EMA are simultaneously expressed in the majority of patients. CD34 foci are positive in individual cases, while keratin, CK, and S-100 proteins are negative [13].

PPM mostly has a good prognosis, without recurrence and metastasis [14]. The main strategy for treatment is surgical resection of the lung, and wedge resection or lobectomy for a benign PPM is usually performed. However, considering the report by Satoh *et al*., which presented the 20-year follow-up findings of remnant PPM lesions exhibiting slow growth with a doubling time of 1393 days, it is imperative to consider the long-term follow-up of several years [15]. Malignant PPMs with aggressive growth and distant metastases are extremely rare. However, there are also reports of malignant PPMs. Prayson *et al*. reported a very aggressive case of PPM. Ipsilateral lobe metastases were detected in a patient 6 months after surgery [16].

In conclusion, PPM could have a variety of radiological findings. As there are no specific radiologic features for the diagnosis of PPM, complete resection of the lesion is required for both diagnosis and treatment. It is necessary to note the imaging features of PPM, presenting as a GGN; this rare tumor should be considered in differential diagnoses.

## Data Availability

All patient records, operation notes, and radiographic information are available in the form of hard copies. Scanned documents can be provided upon request from the journal.

## Abbreviations

PPM: Primary pulmonary meningioma
GGN: Ground glass nodule
CT: Computed tomography
CNS: Central nervous system
EMA: Epithelial membrane antigen
MRI: Magnetic resonance imaging
